# Q20+ Nanopore sequencing data recover a high-quality *Asticcacaulis* sp. genome

**DOI:** 10.1128/MRA.00717-23

**Published:** 2023-09-29

**Authors:** Cyrill Hofer, Seraina Lingenhag, Lucas Serra Moncadas, Angel Rain-Franco, Adrian-Stefan Andrei

**Affiliations:** 1 Department of Plant and Microbial Biology, Limnological Station, University of Zurich, Kilchberg, Switzerland; 2 Faculty of Science, University of Zurich, Zurich, Switzerland; DOE Joint Genome Institute, Berkeley, California, USA

**Keywords:** genomics, DNA sequencing, Nanopore, genomes, bacteria, Asticcacaulis

## Abstract

We present here the complete genome of *Asticcacaulis* sp. ZE23SCel15. The strain was isolated from the surface water of Lake Zurich, Switzerland. The assembly of high-quality Q20+ Nanopore data yielded a circular genome with ~3.8 Mb (coverage: 34×) and a GC content of 56.81%.

## ANNOUNCEMENT

The few described members of the genus *Asticcacaulis* are stalked, motile bacteria that received their generic name due to the non-sticking nature of their prosthecum (1, 2). They are Gram-negative, affiliated with the Caulobacteraceae family, and mainly found in the surface layers of freshwater ecosystems, aerated soils, and plant roots (2).

The *Asticcacaulis* strain ZE23SCel15 was isolated from the surface water of Lake Zurich (47°18*'*N–8°34*'*E, 15 May 2023, Switzerland). Briefly, water was collected and incubated at 18°C in agar plates spiked with cellobiose (1 mM) for 3 weeks in the dark, purified by multiple transfer cycles, and cryopreserved (3). The obtained strain was grown in artificial lake water (4) with glucose as carbon source (100 µM) prior to proliferation in LB (Difco, 240210). DNA was extracted using the Quick-DNA HMW MagBead Kit (Zymo Research) before sample cleanup using the Agencourt AMPure XP beads (Beckman Coulter). To prepare the sequencing library, the Native Barcoding Kit 24 V14 (SQK-NBD114-24, Oxford Nanopore) was used with 1 µg of pure (A260/280 = 1.877, A260/230 = 2.314) high molecular weight (~50 kb, GeneRuler High Range DNA Ladder—Thermo Scientific) DNA as input. The FLO-MIN114-R10.4.1 flow cell was loaded with 5 fmol (154.5 ng for 50,000 bp fragments, NEBioCalculator v1.15.4) of sample and sequenced on the minION-Mk1B (Oxford Nanopore) before basecalling with guppy v.6.4.6 (basecalling model: super accurate basecalling, 400 bps). The sequencing run yielded 683,622 reads with a mean quality of Q13.6 (~95.63% accuracy) and an N50 of 11,344 bp. The data were filtered with chopper (v.0.2.0) to remove reads below Q17 and shorter than 1,000 bp, retaining 36,941 sequences with a mean quality score of 17.9 (~98.38% accuracy) and an N50 of 9,991 bp. Without any further correction, the reads were assembled with Flye (v.2.9.2-b1786) choosing the high-quality read flag for data with less than 3% average error (flye --nano-hq --meta). A circular chromosome (99.35% complete, 0% contamination, CheckM v.1.2.2) with a length of 3,774,217 bp and a GC content of 56.18% was recovered. The whole genome was classified with GTDB-Tk (v.2.2.6) against the Genome Taxonomy Database release 214.1 (5). The 16S rRNA genes, extracted with barrnap (v.0.9), were classified using the SILVA 138.1 SSU (6) and NCBI (7) databases. The predicted genes (Prokka v.1.12) were annotated using PFAM (8), KEGG (9), MetaCyc (10), and NCBI (7).

The closest assembled genome in GTDB (5) is *Asticcacaulis* sp. UBA2476 (GCA_002342035.1), showing 93.55% genomic similarity with a coverage of 88%, isolated from Lake Michigan in the United States. The closest described species regarding 16S rRNA similarity was found to be the plant root-associated *Asticcacaulis endophyticus* [NR_133971.1, NCBI (7)] with 100% sequence similarity (coverage:92%). However, the reference genome of *Asticcacaulis endophyticus* (GCF_014652575.1) is only 92.57% similar with a coverage of 82.51%. Therefore, we show that the isolated strain *Asticcacaulis* sp. ZE23SCel15 possibly belongs to a yet undefined *Asticcacaulis* species. The examination of the 3,430 identified proteins revealed that this strain likely catabolizes sugars via the Entner-Doudoroff pathway, contains two 16S rRNA operons, seems to prefer an aerobic environment where it utilizes the TCA cycle and oxidative phosphorylation, and appears to invest in the assembly of a flagellum, indicating its copiotrophic, aerobic, and heterotrophic lifestyle in aquatic environments.

## Data Availability

The underlying data as well as the complete genome and further information can be found at NCBI via BioProject PRJNA991734 (Accession: CP130044, BioSample: SAMN36315994, SRA: SRR25177533).
